# Mindfulness-based intervention for teenagers with cancer: study protocol for a randomized controlled trial

**DOI:** 10.1186/1745-6215-14-135

**Published:** 2013-05-10

**Authors:** Catherine Malboeuf-Hurtubise, Marie Achille, Serge Sultan, Majorie Vadnais

**Affiliations:** 1 Université de Montréal, Montreal, Quebec, Canada; 2 CHU Sainte-Justine, Mother and Child University Hospital Center, Montreal, Quebec, Canada; 3 Université de Montréal, Pavillon Marie-Victorin, Local D-325, 90 avenue Vincent-d’Indy, Montreal, Québec, H2V 2S9, Canada

**Keywords:** Mindfulness-based meditation, Pediatric cancer, Quality of life, Mood, Sleep, Adolescence, Health psychology

## Abstract

**Background:**

Individuals living with cancer must learn to face not only the physical symptoms of their condition, but also the anxiety and uncertainty related to the progression of the disease, the anticipation of physical and emotional pain related to illness and treatment, the significant changes implied in living with cancer, as well as the fear of recurrence after remission. Mindfulness-based meditation constitutes a promising option to alleviate these manifestations.

**Methods/Design:**

This article presents the rationale and protocol development for a research project aimed at evaluating the effects of a mindfulness-based meditation intervention on quality of life, sleep, and mood in adolescents with cancer compared to a control group. A prospective, longitudinal, experimental design involving three time points (baseline, post-intervention, and follow-up) and two groups (experimental and control) was developed for this project. Participants will be assigned randomly to either group. Eligible participants are adolescents aged 11 to 18 years with a diagnosis of cancer, with no specific selection/exclusion based on type, stage, or trajectory of cancer. A final sample size of 28 participants is targeted. Adolescents in the experimental group will be completing the mindfulness meditation intervention, taught by two trained therapists. The intervention will comprise of eight weekly sessions, lasting 90 min each. Once the follow-up assessment is completed by the experimental group, wait-list controls will be offered to complete the mindfulness-based program. Intra-group analyses will serve to evaluate the impact of the mindfulness-based meditation intervention on quality of life, sleep, and mood pre-post intervention, as well as follow-up. Analyses will also be used to carry out inter-group comparisons between the experimental group and the wait-list controls. Voluntary participation, risk of attrition, and the small sample size are potential limitations of this project. In spite of possible limitations, this project will be one among very few aimed at improving quality of life, sleep, and mood in adolescents living with cancer, will evaluate the potential benefits of such a practice on both psychological and physical health of youth with cancer, and help in creating mindfulness-based intervention programs, in order to provide the necessary psychological help to adolescents living with cancer.

**Trial registration:**

Trial registration number: NCT01783418

## Background

News of a cancer diagnosis has been said to induce the greatest psychological distress among all prognoses and illnesses [1]. Thus, it often elicits a strong emotional reaction in teenagers and their parents [2]. Individuals living with cancer must learn to face not only the physical symptoms of their condition, but also the anxiety and uncertainty related to the progression of the disease, the anticipation of physical and emotional pain related to illness and treatment, the significant changes implied in living with cancer (for example, hair loss, frequent visits to the hospital), as well as the fear of recurrence after remission [3,4]. To address these challenges, various psychosocial approaches have been developed, including mind-body therapies. Mindfulness-based meditation, initially developed to improve quality of life in patients suffering from chronic illnesses, constitutes a promising option [5]. The goal of this article is to describe the development, rationale, and first phase of a pilot study evaluating the effects of a mindfulness-based intervention on quality of life, mood, and sleep for teenagers with cancer.

### The mindfulness-based stress reduction program

Mindfulness can be defined as the process by which we ‘[…examine] who we are, with questioning our view of the world and our place in it, and […cultivate] some appreciation for the fullness of each moment we are alive. Most of all, it has to do with being in touch.’ [6]. It lies at the center of the Minfulness-Based Stress Reduction program (MBSR) developed by Kabat-Zinn and colleagues [5]. Specifically, mindfulness meditation allows us to ‘[…pay] attention in a particular way: on purpose, in the present moment, and nonjudgmentally’.

Kabat-Zinn and colleagues [5] initially developed the MBSR program to address, explore, and decrease emotional and physical pain in patients suffering from a chronic illness, while observing the connection between the mind and the body. Anxiety and worry fueled by past and future events are hypothesized to ruin both physical and psychological health by increasing levels of stress. Thus, the MBSR program aims to bring focus to the present moment [7]. Its main goal is to bring awareness to different manifestations of stress, in order to detect first signs of anxiety and to act voluntarily and willingly to correct usual patterns of response. Thus, the anxiety and depression that often accompany stress can be targeted quickly, in order to achieve general wellbeing and peace of mind [8].

### Literature review and rationale

As previously mentioned, individuals living with cancer are at risk for significant psychological distress [3,4,9]. Approximately 20% to 30% of individuals with cancer manifest depressive symptoms of clinical significance [10]. Moreover, chronic physical symptoms, such as sleep disorders and fatigue, tend to appear consequently to cancer treatments; approximately 85% of individuals with cancer suffer from sleep disorders [11,12].

Recent studies have shown a positive impact of MBSR on psychological health in individuals with cancer. Specifically, a recent meta-analysis has indicated moderate effect sizes of mindfulness meditation on stress, anxiety, depression, quality of life, sleep, and fatigue related to cancer, highlighting the predominant role this practice can play in the individual’s psycho-social adaptation [9]. Research has also shown both immediate and 1-year post-intervention positive impacts of the practice on stress, quality of life, and mood in the same population [11,13,14]. Furthermore, the group setting in which MBSR takes place has been shown to decrease isolation and loneliness often observed in the context of cancer, therefore promoting better relationships and stronger social support, which, in turn, may explain improvements in mood and quality of life [15].

Altogether, authors have concluded that mindfulness meditation has beneficial effects on physical and psychological symptoms among adults with different chronic illnesses, and could hence be a key tool in the treatment of these conditions [16,17]. Emerging research in pediatrics is suggesting similar effects in children and adolescents [18-21]. The few studies that have examined its effects on youth with chronic illnesses tend to suggest that mindfulness meditation acts similarly by decreasing physical and emotional pain [22].

### Primary aim

This article presents the rationale and protocol development for a research project aimed at evaluating the effects of a mindfulness-based meditation intervention on quality of life, sleep, and mood in adolescents with cancer compared to a control group. In light of data available on adult populations and emerging from research in youth, three hypotheses are formulated: (1) mindfulness meditation will have beneficial effects on the mood of adolescents completing this practice; (2) mindfulness meditation will have beneficial effects on quality of life of the participants; and (3) mindfulness meditation will have beneficial effects on sleep quality of the participants.

Specifically, significant improvements are expected to be observed in quality of life, mood, and sleep between pre-intervention and post-intervention measures in the experimental group. These effects should be sustained 6-months post-intervention.

## Methods/Design

### Design overview

**Figure 1:**
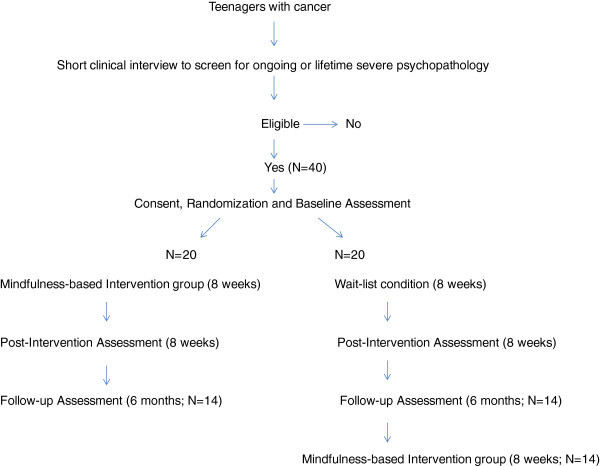
Design and subject flow.

A prospective, longitudinal, experimental design involving three time points (baseline, post-intervention, and follow-up) and two groups (experimental and control) was developed for this project. Participants will be assigned randomly to either the experimental or control group. The longitudinal design will allow the evaluation of the short- and long-term effects of the intervention. Figure 1 illustrates the overall design and subject flow for this project. All study procedures and consent forms received approval from both the Université de Montréal (certificate number CERFAS-2012-13-007-D) and Sainte-Justine Mother and Child University Hospital Center (file number 3550) ethics and scientific committees.

### Participants

Based on prior studies testing cognitive-behavioral interventions in adolescents with cancer, eligible participants are adolescents aged 11 to 18 years with a diagnosis of cancer [23-25]. In keeping with previous research, there will be no specific selection/exclusion based on type, stage, or trajectory of cancer [11,26-28]. The intervention will be conducted in French. Therefore, participants are required to speak and understand the language. They must be willing to participate in an 8-week mindfulness meditation program and be available to complete the measures 6 months after the intervention. They must not have a diagnosis of severe mental illness.

Based on prior mindfulness intervention studies with adolescent populations and on usual number of participants in a mindfulness-intervention training group (as indicated by Kabat-Zinn and colleagues [29] in the MBSR program) a final sample size of 28 participants is targeted [13,29-33]. Taking into consideration attrition rates from prior research (20-30%), an additional 12 participants will be recruited [2,34-36]. Thus, a total of 40 participants will be recruited. Participants will be randomly assigned to the experimental or control group, and immediately given a participant ID for confidentiality and anonymization purposes. Twenty adolescents will be completing the mindfulness meditation intervention, which will be taught by two trained therapists (doctoral clinical psychology student and psychiatrist). The intervention will comprise of eight weekly sessions, lasting 90 min each.

### Recruitment

Recruitment for this project has started in September 2012, and is performed in the oncology center of a university-affiliated pediatric hospital in a large urban area. Physicians and nurses have been invited to discuss the project with their patients when they visit the hospital for their regularly scheduled appointments. In addition, research nurses have screened hospital records to identify patients who qualify for this study (that is, teenagers with cancer and no ongoing or lifetime severe psychopathology, such as schizophrenia, psychosis, delusional disorders, or organic mental disorders), and introduced to them the project. Posters and brochures have been posted and available throughout the oncology wing of the hospital. Supplemental advertisement has been sent through the province-wide Association for Children with Cancer’s monthly newsletter, via mass emailing.

### Screening procedures, consent to participate, and enrollment

One of two screening and enrollment procedures has been applied, depending on the modality of first contact with potential candidates for the project. When contacted by telephone by teenagers having heard of the project and interested in participating, researchers summarize the study, and a meeting is scheduled to complete screening procedures, sign the consent forms, and complete pre-intervention measures. When participants are recruited directly on site, screening, consent, enrollment, and pre-intervention measures are completed immediately. Participants are informed of the possibility of a delay (a few weeks up to a few months) before starting the intervention, as participants continue to be recruited. Informed consent will be obtained by all recruited participants. Intervention will begin either when: (1) 20 participants are selected to be in the experimental group; or (2) 10 participants are selected to be in the experimental group (thus implying that two experimental groups of 10 participants will be run), depending on recruitment speed and efficiency.

### Randomization

Participants will be assigned to one of two groups according to a computer-generated randomization list (computerized random numbers). As such, participants will be randomly assigned with a 1:1 allocation ratio following permuted block design procedures, using block sizes of four. No stratification criteria will be used. The allocation sequence will be generated by an independent researcher not involved in this project after baseline measures, thus ensuring that therapists and recruiters are kept blind to the allocation of each participant. The allocation sequence will be password protected and only accessible by the independent researcher. The research assistant responsible for the logistics of this project - who is not part of the treatment team - will not be kept blind for allocation and will be responsible for contacting participants randomly assigned to the experimental condition.

### Wait-list

The use of a wait-list control condition has been compelling in past studies on MBSR and cancer to test for the effectiveness of the intervention without compromising recruitment, while favoring retention of participants who will later benefit from the intervention [36,37]. Once the follow-up assessment has been completed with the experimental group, wait-list controls will be offered to complete the mindfulness-based program. The data collected on these participants through the second round of intervention will also be analyzed to test the mindfulness-based intervention.

### Experimental group

The present mindfulness-based meditation intervention is inspired by protocols designed at the Centre de consultation psychologique specialisé of the Université Catholique de Louvain (Belgium) [38], as well as by Biegel and colleagues [39]. The intervention will be led by two trained therapists (psychologist and psychiatrist), alternating roles (leader *vs.* co-leader) at each session, leading a total of four sessions each. In accordance with the initial MBSR program designed by Kabat-Zinn and colleagues [5], the mindfulness-based intervention will last 8 weeks, the group meeting once a week and each session lasting 90 min. Based on previously cited protocols designed for youth, no silent retreat will be included in this intervention.

### Mindfulness-based intervention protocol

#### Week 1

The mindfulness intervention will begin with an overview of class rules and the 8-week program. Participants will present each other in dyads, then to the whole group. They will also be asked to write down expectations and intentions in regards to the intervention (these will be collected by the therapists and handed back to all participants at the last session). An introduction to mindful eating with a cranberry tasting exercise will take place. Home practice for this session will include completing mindful tasks during the week and a ‘thinking outside the box’ exercise. A minimal practice of 30 min each week, followed by an entry in their weekly meditation journal will be suggested to participants.

#### Week 2

Participants will be guided through a body scan meditation. An introduction to the different components of emotions (that is, thoughts, physical sensations, behavior) and stress, as well as their effect on the body and mind will be discussed. An outstretched arms meditation will be completed, in order to practice mindfulness through pain. Home practice will include familiarization with the body scan and cultivating mindfulness of the body.

#### Week 3

Participants will be guided through a breathing meditation and will be introduced to sitting meditation. Mindful movements will also be introduced, through gentle yoga-like poses inspired by readings from *Mindful Movements: Ten Exercises for Well-Being*[40]. Home practice will include sitting meditation and mindful breathing in the context of pleasant and unpleasant events.

#### Week 4

Participants will be guided through a breathing meditation. Concepts of acceptance of thoughts, emotions, and bodily sensations will be discussed. In dyads, participants will be asked to exchange about suffering. During this activity, they will be asked to stop periodically and do a mindful check-in of how they are feeling while discussing a difficult topic. Home practice will include sitting meditation and the reading of Rumi’s *The Guest House* poem.

#### Week 5

Participants will be guided through a breathing meditation, with a special focus on thoughts and judgments. Mindful senses will then be explored, mainly through mindful touching and breathing. Participants will complete an inhibition activity and will be asked to reflect on our natural propensity to have spontaneous negative thoughts and judgments and discussing how we can come to inhibit such thoughts. Home practice will include meditation on thoughts and judgments.

#### Week 6

Participants will be guided through a heartfulness meditation, with a special focus on self-care and sending compassion to others. A group discussion on the topic of self-care *vs.* selfishness will take place. The concept of acceptance will also be explored. The session will end with a short sitting meditation with an emphasis on acceptance. Home practice will include heartfulness meditation and mindful activities that provide self-care.

#### Week 7

Participants will be guided through the mountain meditation. In dyads, participants will be asked to recall an autobiographical memory and identify how this memory reflects their personal values. A group discussion on personal and life values will then take place. Participants will be asked to reflect whether mindfulness can be considered as a value or a tool in living in accordance with our personal values. Home practice will include any type of meditation practiced in the previous weeks, as well as a reflecting on the importance of being mindful while acting accordingly to personal values.

#### Week 8

Participants will have the choice of the type (sitting *vs*. lying down) of meditation and special focus they want to bring to the practice. Intentions set at the first session will be redistributed to all participants. Participants will be given the opportunity to exchange about the intervention and give comments and feedback to the therapists. A giant puzzle activity will take place, in which participants will be asked to write on each piece personal benefits and/or disadvantages of the intervention and their meditation practice. The session will end with the distribution of a pebble stone to each participant as a reminder of their experience.

### Measures

#### Assessment time-points

**Figure 2:**
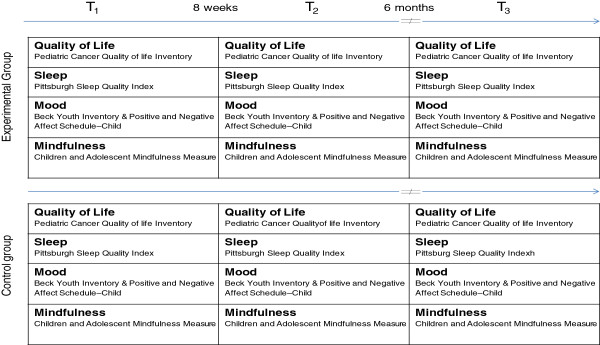
Measures and assessment time points.

Measures will be completed by all participants (that is, experimental and control group) at three moments in time: (1) baseline, after the screening interview, consent forms are signed and enrollment is confirmed; (2) immediately post-intervention, at the end of the eighth session; and (3) at 6 months follow-up, when questionnaires will be sent out by mail. At each time period, participants will fill out the questionnaire package, taking approximately 60 min to complete. There will be time allocated at the first and last sessions to complete questionnaires on site. After the third assessment period, once the last questionnaire package has been sent back to the research team, a $10 iTunes gift cards will be mailed to each participant to thank him/her for his/her participation. All measures and corresponding assessment time-points are illustrated in Figure 2.

### Primary outcome measures

Beck Youth Inventories™ - Depression and anxiety scales [41]: this measure will be used to evaluate symptoms of depression and anxiety. Each scale comprises 20 items, for which participants are asked to indicate on a Likert-type scale ranging from 0 (‘never’) to 3 (‘always’) how frequently each statement applied to them in the past two weeks. Both scales have presented excellent internal consistency (α >0.84) and good reliability (r >0.73) for adolescent populations [42]. For the purposes of this project, a validated French version will be used [43].

Positive and Negative Affect Schedule - Child (PANAS-C) [44]: this measure is used to evaluate the presence and fluctuations in positive and negative emotions. The questionnaire comprises 20 items, for which participants are asked to answer on a Likert-type scale ranging from 1 (‘not much or not at all’) and 5 (‘a lot’), whether the described emotion matches what they have felt like in the past few weeks. This measure presents excellent results in terms of internal consistency (α ranging from 0.91 to 0.92) and good reliability for adolescent populations [45]. A French version has been validated, and will be used for this project [46].

Pediatric Cancer Quality of Life Inventory [47]: this measure is used to evaluate quality of life in children and adolescents with cancer. The questionnaire comprises 27 items for which participants are asked to indicate on a Likert-type scale ranging from 0 (‘never’) to 4 (‘almost always’) how much each symptom has been problematic in the past month. The measure has good to excellent psychometric properties in terms of construct validity, reliability, internal consistency (α = 0.91 to 0.92) and clinical validity [47]. A French version of this questionnaire is available and will be used for this project [48].

Pittsburgh Sleep Quality Index [49]: this measure evaluates sleeping habits in the past month. The questionnaire comprises 11 items, and presents good psychometric properties in terms of internal consistency (α = 0.83), test-retest reliability (r = 0.85), and convergent validity [49]. This measure is commonly used with adolescents and is validated in French [50-52].

### Process measure

Children and Adolescent Mindfulness Measure [53]: this measure is used to evaluate mindfulness in children and adolescents. The questionnaire comprises 25 items, for which participants are asked to indicate on a Likert-type scale ranging from 0 (‘never true’) to 4 (‘always true’) how often each item is true for them. This measure is included in the project in order to assess the extent to which adolescents become more mindful as they receive the intervention, as well as to control for existing differences between participants in their natural tendency to embody mindfulness concepts. The measure presents good internal consistency ( α = 0.87), as well as good convergent validity, and is available in French [53].

In addition, in-house social support analog scales presently in development will be administered, in order to evaluate if any improvements on quality of life, mood, and sleep can be linked to social support and the overall social nature of the intervention.

### Planned statistical analyses

Descriptive statistics on participant characteristics will be presented for each group at baseline. Means and standard deviations on primary and secondary outcome variables will be presented for each group at 8 weeks and 6 months.

The cut-point to reject the null hypothesis will be set at 5% (α ≤0.05). Primary hypotheses will first be tested using three mixed analyses of covariance (ANCOVA) with Bonferonni corrections and individual critical alpha levels at 1.67% (α ≤0.0167), that will allow comparison of data at each time-point [54]. The use of mixed ANCOVAs will help reduce within-group error variance, namely by controlling for baseline differences between participants in each condition and by taking into account possible confounds (for example, age, diagnosis, cancer stage), while providing a higher statistical power in detecting an intervention effect ( [55,56]).These intra-group analyses will serve to evaluate the impact of the mindfulness-based meditation intervention on quality of life, sleep, and mood pre-post intervention, as well as follow-up.

Specifically, changes in quality of life scores (as indicated by the Pediatric Cancer Quality of Life Inventory) between baseline, 8 weeks and 6 months will be tested. Similarly, a change in sleep quality scores (as indicated by the Pittsburgh Sleep Quality Index) and mood scores (as indicated by the Depression and anxiety scales of the Beck Youth Inventories and the PANAS-C) will be tested between baseline, 8 weeks and 6 months. These analyses will also serve to test secondary hypotheses, by allowing inter-group comparisons between the experimental group and the wait-list controls. Specifically, a difference in quality of life scores, sleep quality scores, and mood scores at baseline, 8 weeks, and 6 months will be assessed between groups.

Comparable analyses will be conducted on the data collected from the second experimental group once participants from the wait list condition go through the intervention. All statistical analyses will be conducted using SPSS version 20 (SPSS Inc., Chicago, IL, USA).

### Statistical power

Effect sizes will first be calculated using partial eta squared (partial η^2^) in order to evaluate the pre-post effect of the intervention. Cohen’s d will also be calculated in order to evaluate the effect of the intervention between groups. Based on the previous literature on MBSR and oncology, small effect sizes are usually found [36]. Given the pilot-testing nature of this study, it may be that we will not detect large group differences. In this perspective, clinical significance and cues of intervention success will be evaluated based on individual courses and established cut-points for each primary outcome variable. Participant journals may also be taken into consideration for qualitative information regarding the intervention.

### Quality control

Therapists will keep a weekly log of each session, including attendance, level of participation, activities that were preferred, not correctly understood, or not appreciated by participants. All participants will be encouraged to keep a mindfulness diary, in which information such as impressions, home practice, formal and informal meditations, time spent meditating in the past week, hospital visits, sick days, and general wellbeing could be logged in. Videotaping of each session will ensure intervention validity, as both therapists will receive 1 h of clinical supervision for each session, provided by two duly trained MBSR and mindfulness-based cognitive therapy (MBCT) psychologists from the Centre de consultation psychologique specialisé of the Université Catholique de Louvain (Belgium) who will review the videos.

### Pilot study endpoints

Based on previous research, the following criteria will be evaluated in order to assess the feasibility of a subsequent, larger trial [57]:

1. Recruitment and retention: Recruitment effectiveness will be closely monitored and documented, along with recruitment strategies. Ineffective strategies will be eliminated in subsequent trials. Retention and attrition rates will also be taken into consideration in developing further trials, especially in regards to future estimates of sample sizes and inclusion/exclusion criteria, if relevant.

2. Feasibility and design assessment: Notes will be taken regarding design flow and overall progress of this project, namely the time frame required to complete each step planned in this design and the overall study.

3. Intervention and measure assessment: Participants will be asked to provide feedback to the therapists regarding their appreciation of the mindfulness intervention. They will also be asked if they would recommend this intervention to peers with cancer. Measure assessment and acceptability will be evaluated according to rates of completion of each questionnaire after the last assessment point. Thus, measures with low completion rates or poor understandability will be reconsidered for subsequent, larger trials.

4. Outcome assessment: Statistical analyses will provide indications of potential beneficial effects of the mindfulness intervention, namely with effect sizes as indicators of the magnitude of observed improvements on mood, sleep, and quality of life. These results could help in evaluating the relevance of including these variables in further trials.

## Discussion

Voluntary participation to this project constitutes a potential limitation, as it could imply that adolescents and their parents who take part are individuals who are either: (1) genuinely interested in the concept of mindfulness and in the action of meditating, thus potentially displaying a higher level of mindfulness than the general population of adolescents with cancer; or (2) potentially healthier and in better shape than those who chose not to participate, as meditation can be demanding. In both cases, these potential confounds will likely limit our ability to generalize our results primarily to adolescents with cancer showing similar motivation and level of health.

We do expect recruitment challenges, specifically in terms of presenting our research to ill adolescents and their parents, convincing them to take part in this project, and the fact that pediatric cancers are less frequent than adult cancers. However, we will maximize recruitment by using multiple recruitment strategies and different avenues to publicize this study.

The risk for attrition is an additional limitation of this project. Given the target population for the study, it is possible that attrition will be observed during the process due to diverse reasons, namely deterioration of health, initiation, or reinitiation of treatment rounds. Drop-outs could also occur due to the challenging nature of the intervention. Participants may conclude that the program is not what they expected or is simply too demanding for them. Further drop-outs may occur 6 months post-intervention, as some participants may be lost to follow-up (for example, harder to reach, no longer motivated to participate in the project). In addition, the research team is weighing out different options to minimize attrition in the wait-list control group - namely, monetary compensation and/or additional iTunes gift cards.

In spite of possible limitations, this project will be one among very few aimed at improving quality of life, sleep, and mood in adolescents living with cancer. To this day, mindfulness meditation has not been empirically tested with youth suffering from this condition. This project will help in evaluating the potential benefits of such a practice on both psychological and physical health of youth with cancer. Moreover, as psychological support programs in pediatric oncology are rare, conclusive results from this project could help in creating mindfulness-based intervention programs, in order to provide the necessary psychological help to adolescents living with cancer.

## Trial status

This is a clinical trial with ongoing patient recruitment. Recruitment for this project started in September 2012, and is expected to be completed by May 2013. This trial is recorded under the number NCT01783418.

## Abbreviations

MBCT: Mindfulness-based cognitive therapy; MBSR: Mindfulness-based stress-reduction

## Competing interests

The authors declared that they have no competing interests.

## Authors’ contributions

CMH, MA, and SS contributed to the design and conception of this study. CMH and MV have elaborated the intervention protocol and participated in the recruitment of participants. CMH drafted the manuscript. All other authors have contributed to the revision of the initial manuscript and read and approved the final version of the submitted article.
